# Supplementary material for: Deep learning algorithms reveal increased social activity in rats at the onset of the dark phase of the light/dark cycle
Source: PLoS One. 2024 Nov 8;19(11):e0307794. doi: 10.1371/journal.pone.0307794 (PMC11548743; doi:10.1371/journal.pone.0307794)
Supplement: S1 File — (DOCX) [file pone.0307794.s001.docx]

# Supplementary files

Supplementary files including python’s scripts are available at: [https://doi.org/10.17605/OSF.IO/MUQZ2](https://osf.io/muqz2/)

# STAR Methods

| **REAGENT or RESOURCE** | **SOURCE** | **IDENTIFIER** |
| --- | --- | --- |
| **Deposited data** |  |  |
| Raw and analyzed data | This paper | [https://doi.org/10.17605/OSF.IO/MUQZ2](https://osf.io/muqz2/) |
| Python scripts | This paper | [https://doi.org/10.17605/OSF.IO/MUQZ2](https://osf.io/muqz2/) |
| Sample videos | This paper | [https://doi.org/10.17605/OSF.IO/MUQZ2](https://osf.io/muqz2/) |
| **Experimental models: Organisms/strains** |  |  |
| Sprague-Dawley Norway Rats | Charles River, Germany | Crl:CD(SD) |
| **Software and algorithms** |  |  |
| Python, versions 3.6, 3.8.16, 3.9.15, 3.12 | Python Software Foundation | https://www.python.org |
| iPython, version 8.6.0 | Python Software Foundation | https://ipython.org |
| MedPC-IV, version SOF-735 | Med Associates | https://med-associates.com/product/med-pc |
| DeepLabCut, version 2.3rc2 | The Mathis Lab of Adaptive Intelligence | https://github.com/DeepLabCut |
| DeepLabCut, version 2.3.5 GUI | The Mathis Lab of Adaptive Intelligence | https://github.com/DeepLabCut |
| SimBA, version 1.87 GUI | The Golden Lab | https://github.com/sgoldenlab/simba |
| MatLab, version: R2023a | MathWorks | https://www.mathworks.com/products/matlab.html |
| DeepSqueak, version 3.1.0 | Coffey et al., 2019 | https://github.com/DrCoffey/DeepSqueak |
| **Other** |  |  |
| Video Cameras | Axis | Model: M1137 Mk II <https://www.axis.com/products/axis-m1137-e-mk-ii> |
| Microphones | Avisoft-Bioacoustics | Model: CM16/CMPA |
| Ultra Sound Gate | Avisoft-Bioacoustics | Model: 416H |
| Water bottle + Sipper Tube | Med Associates | Model: ENV-250BT |
| Head Entry Detector | Med Associates | Model: ENV-254-CB |
| Fans | Sunon | Model: MagLev MF80251V1-1000U-G99 12V DC 1,44W DC |
| LED Light | Polux | Model: NEON 2 m 17W 680 lm 4000K IP65 |

Scripts used in this work are available at [https://doi.org/10.17605/OSF.IO/MUQZ2](https://osf.io/muqz2/)

01-popik_et_al_20240629.py

02-popik_et_al_20240629.py

03-popik_et_al_20240629.py

04-popik_et_al_20240629.py

05-popik_et_al_20240629.py

06-popik_et_al_20240629.py

07-popik_et_al_20240629.py

08-popik_et_al_20240629.py

09-popik_et_al_20240629.py

10-popik_et_al_20240629.py

11-popik_et_al_20240629.py

12-popik_et_al_20240629.py

13-popik_et_al_20240629.py

14-popik_et_al_20240629.py

15-popik_et_al_20240629.py

16-popik_et_al_20240629.py

17-popik_et_al_20240629.py

18-popik_et_al_20240629.py

19-popik_et_al_20240629.py

20-popik_et_al_20240629.py

21-popik_et_al_20240629.py

22-popik_et_al_20240629.py

23-popik_et_al_20240629.py

24-popik_et_al_20240629.py

25-popik_et_al_20240629.py

26-popik_et_al_20240629.py

27-popik_et_al_20240629.py

28-popik_et_al_20240629.py

29-popik_et_al_20240629.py

30-popik_et_al_20240629.py

31-popik_et_al_20240629.py

32-popik_et_al_20240629.py

33E-popik_et_al_20240629.py or 33M-popik_et_al_20240629.py

34E-popik_et_al_20240629.py or 34M-popik_et_al_20240629.py

35E-popik_et_al_20240629.py or 35M-popik_et_al_20240629.py
